# Comparison of SGLT2 Inhibitors for New‐Onset Proteinuria Risk in Patients With Type 2 Diabetes and Preserved Kidney Function

**DOI:** 10.1111/dom.70625

**Published:** 2026-03-09

**Authors:** Hiroki Nobayashi, Michihiro Satoh, Takuo Hirose, Shingo Nakayama, Yutaro Iwabe, Hideaki Hashimoto, Takahisa Murakami, Kouji Okada, Takefumi Mori, Hirohito Metoki

**Affiliations:** ^1^ Division of Public Health, Hygiene and Epidemiology, Faculty of Medicine Tohoku Medical and Pharmaceutical University Sendai Japan; ^2^ Department of Pharmacy Tohoku Medical and Pharmaceutical University Hospital Sendai Japan; ^3^ Department of Preventive Medicine and Epidemiology Tohoku Medical Megabank Organization, Tohoku University Sendai Japan; ^4^ Division of Nephrology and Hypertension, Faculty of Medicine Tohoku Medical and Pharmaceutical University Sendai Japan; ^5^ Division of Clinical Pharmaceutics and Pharmacy Practice, Faculty of Pharmaceutical Sciences Tohoku Medical and Pharmaceutical University Sendai Japan

**Keywords:** database research, diabetic nephropathy, pharmaco‐epidemiology, real‐world evidence, SGLT2 inhibitor

## Abstract

**Aims:**

To compare the effects of individual SGLT2 inhibitors on preventing new‐onset proteinuria in patients with type 2 diabetes and preserved kidney function.

**Materials and Methods:**

A target trial emulation was performed using commercially available databases, including health checkups and claims data, between April 1, 2014 and March 31, 2023. Patients with type 2 diabetes (HbA1c ≥ 6.5%, use of antidiabetic drugs, and/or disease codes of type 2 diabetes) and preserved kidney function (estimated glomerular filtration rate [eGFR] ≥ 60 mL/min/1.73 m^2^, urinary protein < 1+ and without past history of chronic kidney disease) were included. We compared the risk of new‐onset proteinuria among new users of empagliflozin, dapagliflozin and canagliflozin using the inverse probability of treatment weighting to adjust for baseline confounders and the inverse probability of censoring weighting to account for loss to follow‐up.

**Results:**

The mean age was 67.5 ± 10.3 years, and 60% were men. The mean HbA1c level and eGFR were 7.56% ± 1.32% and 75.0 ± 8.0 mL/min/1.73 m^2^, respectively. During the median follow‐up of 522 [263–925] days, new‐onset proteinuria occurred in 43, 66 and 48 empagliflozin, dapagliflozin and canagliflozin users, respectively. Dapagliflozin showed a higher risk of new‐onset proteinuria (hazard ratio [HR], 1.64; 95% confidence interval [CI], 1.15–2.33) than empagliflozin, particularly in men (HR, 2.34; 95% CI, 1.53–3.58) (*p* for heterogeneity < 0.01).

**Conclusions:**

Empagliflozin was associated with a lower risk of new‐onset proteinuria compared with dapagliflozin. These findings underscore the importance of individualised SGLT2 inhibitor selection but require confirmation in randomised controlled trials.

## 1 Introduction

Diabetes is a major contributor to chronic kidney disease (CKD) and represents the leading cause of end‐stage kidney disease (ESKD) worldwide [1, 2]. The classic clinical course of diabetic kidney disease involves progressive stages of glomerular hyperfiltration, microalbuminuria, overt proteinuria and a decline in the glomerular filtration rate, eventually leading to ESKD [3]. A large‐scale clinical trial and a prediction model validation study assessing the risk of CKD in patients with type 2 diabetes identified albuminuria as the most significant contributing factor [4, 5]. Consequently, preventing albuminuria in patients with diabetes represents a key therapeutic goal for improving renal outcomes.

Sodium–glucose cotransporter 2 inhibitors (SGLT2is) have significantly transformed the therapeutic landscape of type 2 diabetes. In addition to their glucose‐lowering effects, SGLT2is have demonstrated remarkable improvements in renal outcomes among patients with type 2 diabetes in randomised clinical trials (RCTs) [6, 7, 8, 9]. Meta‐analyses of RCTs have demonstrated their favourable effects on albuminuria compared to controls [10, 11, 12]. However, when considering individual SGLT2is, differences in efficacy have been observed, with empagliflozin showing a greater tendency to reduce albuminuria. These meta‐analyses accounted for differences in populations and protocol settings across trials, making direct comparisons of individual SGLT2is within the same analysis impossible. Nevertheless, their findings suggest potential variability in the albuminuria‐suppressing effects of SGLT2is, underscoring the need for real‐world studies with similar baseline characteristics.

RCTs comparing the effects of individual SGLT2is are unavailable and considered unfeasible. Therefore, we used a target trial emulation (TTE) framework with large‐scale real‐world data to compare the risk of new‐onset proteinuria among patients with type 2 diabetes who received empagliflozin, dapagliflozin, or canagliflozin, all of which have kidney‐protective effects.

## 2 Materials and Methods

### 2.1 Study Design: Target Trial Emulation

We emulated a hypothetical pragmatic randomised trial (target trial), following the Transparent Reporting of Observational Studies Emulating a Target Trial guideline [13]. To eliminate prevalent user bias and drug effects at study entry, we implemented a new‐user design [14]. In this study, we compared three SGLT2is (empagliflozin, dapagliflozin and canagliflozin), using empagliflozin as the reference owing to its highest SGLT2 selectivity. The basic study protocol is shown in Table S1.

### 2.2 Study Population

We used commercially available databases provided by DeSC Healthcare Inc. between April 1, 2014 and March 31, 2023. These databases comprise data from a subset of insurers under three public health insurance systems in Japan that have contracts with DeSC: (i) the Health Insurance Society database, which primarily covers employed individuals and their dependents; (ii) the National Health Insurance database, which mainly includes self‐employed individuals, retirees and the unemployed; and (iii) the Later‐Stage Elderly Healthcare System database, which covers adults aged ≥ 75 years (and those aged 65–74 years with designated disabilities) [15]. These databases comprise annual health checkups and claims data. The age and sex distribution in the database provided by DeSC Healthcare Inc. is generally consistent with that of the National Health and Nutrition Survey, which is considered representative of the Japanese population [16]. We included new users of SGLT2is, defined as patients with no records of SGLT2is prescriptions within 180 days before the index date.

We identified patients with type 2 diabetes and preserved kidney function as those with available baseline and follow‐up data. Type 2 diabetes was defined as an HbA1c level ≥ 6.5%, and/or the use of antidiabetic medications, and/or the presence of international classification of diseases (ICD)‐10 codes for non–insulin‐dependent diabetes mellitus (E11, E110–E119). Patients with ICD‐10 codes for insulin‐dependent diabetes mellitus (E10 and E100–E109) were excluded. Preserved kidney function was defined as an estimated glomerular filtration rate (eGFR) ≥ 60 mL/min/1.73 m^2^, a urinary protein level of < 1+ and the absence of past history of CKD (self‐reported questionnaires and ICD‐10 disease codes).

Follow‐up began on the date of initiation of each SGLT2i and ended at the earliest occurrence of the outcome, the last health checkup date when no subsequent checkup was recorded for more than 2 years after the previous visit, or the final health checkup date within the observation period.

This study was approved by the Research Ethics Committee of the Tohoku Medical and Pharmaceutical University for Life Science and Medical Research. The requirement for informed consent was waived because all data were fully de‐identified before the analysis (approval ID: 2024‐2‐015).

### 2.3 Drug Data

The claims contained data on the prescribed medications, classified according to the generic name and anatomical therapeutic chemical classification (ATC) code of the World Health Organization Collaborating Centre for Drug Statistics Methodology (WHO‐ATC code). The drugs used in the analyses are summarised in Table S2 [17, 18]. Concomitant drugs were defined as medications prescribed on the SGLT2is initiation date or with prescription coverage overlapping that date.

### 2.4 Clinical Data

Annual health checkups in Japan are recommended according to the guidelines of the Japanese Ministry of Health, Labor and Welfare [15, 19]. Data on smoking and drinking habits as well as medical history (heart disease, stroke and kidney disease) were obtained via questionnaires administered during baseline health checkups. Data on age, sex and mortality were extracted from the enrolment registry records. Data on body mass index (BMI), systolic blood pressure (SBP), low‐density lipoprotein cholesterol (LDL‐C), HbA1c, eGFR and urinary protein levels were extracted from health checkup records. eGFR was calculated using the Japanese coefficient‐modified Chronic Kidney Disease Epidemiology Collaboration (CKD‐EPI) equation [20]. Data on history of heart failure (HF), ischemic heart disease, cerebrovascular disease and CKD were extracted from claims data linked to the ICD‐10 codes (Table S3). These clinical measurements were obtained on the date closest to the first SGLT2is prescription within the preceding 365 days (median, 161 days; maximum, 365 days prior to prescription).

### 2.5 Outcomes

The primary outcome measure was new onset proteinuria, defined as ≥ 1+ determined by qualitative dipstick testing. A validation study has reported that a dipstick result of ≥ 1+ had a specificity of 95.4% for detecting microalbuminuria, whereas a result of trace or higher had a specificity of 86.8% [21]. Therefore, in this study, dipstick results of trace or negative were defined as negative. Additional interest included changes in eGFR and HbA1c levels from baseline.

### 2.6 Baseline Covariates for Inverse Probability of Treatment Weighting (IPTW)

The covariates listed in Table 1 were selected, except for the daily SGLT2is dose. Enrolment in the Later‐Stage Elderly Healthcare System was included as a covariate because these individuals constitute a distinct population. Trace proteinuria was included because it could be associated with a higher risk of progressing to ≥ 1+ proteinuria. Baseline comorbidities were identified using self‐reported questionnaires and ICD‐10 disease codes and used separately from the data sources. ICD‐10 codes were used to ascertain treatment intent based on the physician's diagnoses. Baseline medication use (Table S2) was included in the model to adjust for the effects of medications. As dapagliflozin was the first SGLT2i to receive approval for HF treatment in Japan on November 27, 2020, we included the prescription period (before or after the date) as a covariate to account for potential differences in prescription patterns.

**TABLE 1:** Participant characteristics after weighting.

	Weighted					
	Empagliflozin *N* = 2933.8	Dapagliflozin *N* = 2989.3	Canagliflozin *N* = 2908.8	|SMD| Empagliflozin vs. dapagliflozin	|SMD| Empagliflozin vs. canagliflozin	|SMD| Dapagliflozin vs. canagliflozin
Age, year (SD)	67.5 (10.0)	67.6 (10.1)	67.4 (10.1)	0.01	0.00	0.01
Later‐stage Elderly Healthcare System, *n* (%)	713 (25)	768 (26)	722 (25)	0.02	0.01	0.01
Male, *n* (%)	1730 (59)	1783 (60)	1790 (62)	0.01	0.03	0.02
BMI, kg/m^2^ (SD)^a^	25.9 (4.0)	25.8 (3.9)	25.9 (4.0)	0.01	0.00	0.01
SBP, mmHg (SD)	132.4 (15.6)	132.3 (15.7)	132.6 (15.6)	0.00	0.01	0.01
Drinker, *n* (%)^a^	453 (19)	486 (20)	422 (17)	0.03	0.01	0.03
Smoker, *n* (%)^a^	460 (18)	459 (18)	454 (18)	0.00	0.01	0.02
LDL‐cholesterol, mmol/L (SD)	2.95 (0.79)	2.95 (0.79)	2.95 (0.78)	0.00	0.00	0.00
HbA1c, % (SD)	7.52 (1.21)	7.56 (1.28)	7.58 (1.35)	0.02	0.03	0.01
Trace proteinuria, *n* (%)	417 (14)	440 (15)	336 (12)	0.04	0.04	0.01
eGFR, mL/min/1.73 m^2^ (SD)	74.9 (7.8)	75.1 (7.8)	75.2 (7.7)	0.01	0.05	0.05
Self‐reported past history, *n* (%)						
Stroke^a^	107 (4)	93 (4)	66 (3)	0.03	0.02	0.01
Heart disease^a^	281 (11)	266 (10)	257 (10)	0.03	0.02	0.00
ICD‐based past history, *n* (%)						
Cerebrovascular disease	260 (9)	194 (7)	273 (9)	0.01	0.05	0.05
Ischemic heart disease	597 (20)	563 (19)	505 (17)	0.02	0.05	0.02
Heart failure	523 (18)	520 (17)	428 (15)	0.01	0.05	0.04
Prescription before dapagliflozin's heart failure indication	1796 (61)	1803 (60)	1906 (66)	0.01	0.06	0.06
Anti‐hypertensive drugs, *n* (%)						
Dihydropyridine derivative	1171 (40)	1239 (41)	1146 (39)	0.02	0.01	0.02
RASI	1278 (44)	1240 (41)	1240 (43)	0.03	0.01	0.01
MRA	83 (3)	93 (3)	56 (2)	0.01	0.04	0.04
Thiazide diuretics	141 (5)	157 (5)	167 (6)	0.01	0.03	0.01
Loop diuretics	113 (4)	104 (3)	61 (2)	0.01	0.07	0.05
β‐αβ blockers	350 (12)	377 (13)	291 (10)	0.01	0.04	0.05
α blockers	31 (1)	21 (1)	29 (1)	0.03	0.00	0.02
Lipid‐lowering drugs, *n* (%)						
HMG CoA reductase inhibitors	1474 (50)	1498 (50)	1448 (50)	0.00	0.01	0.00
Fibrates	154 (5)	163 (5)	191 (7)	0.01	0.03	0.03
Uric acid‐lowering drugs, *n* (%)						
Preparations inhibiting uric acid production	203 (7)	201 (7)	233 (8)	0.00	0.02	0.03
Preparations increasing uric acid excretion	18 (1)	25 (1)	19 (1)	0.02	0.00	0.01
Dose of SGLT2i, mg/day (SD)	10.3 (2.3)	5.3 (1.2)	99.7 (5.4)			
Anti‐diabetic drugs, *n* (%)						
Biguanide	1293 (44)	1245 (42)	1200 (41)	0.03	0.03	0.00
DPP‐4i	1510 (51)	1607 (54)	1662 (57)	0.03	0.07	0.04
Sulfonylurea	575 (20)	568 (19)	513 (18)	0.01	0.03	0.02
Glinide	166 (6)	147 (5)	185 (6)	0.02	0.02	0.04
α‐glucosidase inhibitors	316 (11)	360 (12)	343 (12)	0.03	0.02	0.00
Thiazolidinedione	217 (7)	191 (6)	244 (8)	0.03	0.02	0.04
GLP‐1RA	52 (1)	54 (2)	31 (1)	0.00	0.04	0.04
Insulin	195 (7)	161 (5)	164 (6)	0.03	0.03	0.01

Abbreviations: BMI, body mass index; DPP‐4i, dipeptidyl peptidase 4 inhibitor; eGFR, estimated glomerular filtration rate; GLP‐1RA, glucagon‐like peptide‐1 receptor agonist; HbA1c, haemoglobin A1c; ICD, international classification of diseases; LDL‐C, low‐density lipoprotein cholesterol; MRA, mineralocorticoid receptor antagonist; RASI, renin–angiotensin system inhibitor; SBP, systolic blood pressure; SGLT2i, sodium‐glucose cotranspoter‐2 inhibitor; SMD, standardised mean difference.

^a^ Missing data: BMI, 3 for the dapagliflozin group. Drinker, 501 for empagliflozin, 547 for dapagliflozin, 497 for canagliflozin; smoker, 410 for empagliflozin, 458 for dapagliflozin, and 404 for canagliflozin; stroke, 358 for empagliflozin, 416 for dapagliflozin, and 404 for canagliflozin; heart disease, 355 for empagliflozin, 416 for dapagliflozin, and 404 for canagliflozin.

### 2.7 Disease Definition Based on ICD‐10 Codes

ICD‐10 codes were used as part of the criteria to classify types 1 and 2 diabetes during patient selection, to define baseline comorbidities and to identify the occurrence of urinary tract infection (UTI) in sensitivity analyses (Table S3). Previous studies have demonstrated acceptable validity of disease identification using the Japanese Diagnosis Procedure Combination data. Case identification showed a positive predictive value (PPV) exceeding 80% for cerebrovascular disease and myocardial infarction [22, 23, 24], whereas validation studies for HF and CKD have reported moderate PPVs of approximately 60% [24, 25]. For non‐insulin‐dependent diabetes mellitus, a PPV exceeding 80% has been reported using a combination of ICD codes and glucose‐lowering medication use [26, 27], an approach adopted in the present study. For insulin‐dependent diabetes mellitus and UTI, diagnostic codes were applied in accordance with previous studies, in the absence of formal validation data [28, 29, 30, 31].

### 2.8 Statistical Analysis

Covariables are summarised as means (standard deviations) and numbers (%), as appropriate. We used IPTW to control for baseline confounding, as mentioned in the previous section [32]. We estimated the probability of receiving empagliflozin, dapagliflozin, or canagliflozin as a function of baseline covariates using a generalised boosted model for propensity score estimation. The IPTW was then derived from the estimated propensity scores to create a weighted pseudo‐population in which the baseline covariates were balanced across the treatment groups. The analysis was performed using SAS, with additional computations conducted in R (version 4.4.2) using the twang package, which implements the generalised boosted modelling approach (via the mnps function) to estimate multivariate propensity scores and the average treatment effect. Weighting was considered appropriate when the absolute standardised mean difference (SMD) between treatment groups was < 0.1 [33]. Missing values for continuous variables were imputed using age‐based regression stratified by sex. Missing categorical variables were handled using a missing‐indicator approach because they pertained only to the covariates used for the estimation of the inverse probability weights and not to the outcome itself.

To account for loss to follow‐up due to non‐attendance at health checkups, individuals were censored at the time of non‐attendance, and the inverse probability of censoring weighting (IPCW) was estimated using time‐varying covariates, including age, BMI, SBP, LDL‐C, HbA1c, eGFR and trace proteinuria, with weights truncated at the 99th percentile [34]. Final weights were calculated as the product of IPTW and IPCW. Weighted pooled logistic regression models, including treatment group and follow‐up time, were then fitted to estimate absolute risk and risk difference at 3 years for new‐onset proteinuria, corresponding approximately to the third quartile of the observation period [35]. This approach also provides estimates equivalent to hazard ratios (HRs) and 95% confidence intervals (CIs) based on a Cox model [36]. Effect estimates, including weighted HRs, absolute risks and risk differences, along with their 95% CIs, were obtained from 1000 bootstrap replications. In the primary analyses, we employed an intention‐to‐treat approach, analysing patients according to their initially assigned treatment group, regardless of discontinuation or switching. Changes in eGFR and HbA1c from baseline were evaluated for up to 3 years after treatment initiation, with weighted mean changes and corresponding CIs at 3 years estimated using bootstrap resampling.

We conducted subgroup analyses according to age (70 years, ≥ 70 years), sex (male, female), BMI (< median 25.4 kg/m^2^, ≥ 25.4 kg/m^2^), HbA1c level (< median 7.3%, ≥ 7.3%) and use of renin‐angiotensin‐aldosterone system (RAAS) inhibitors. Interactions between the subgroups were evaluated using heterogeneity tests based on the Q statistics derived from the model. Heterogeneity with *p* < 0.05 indicated significant interaction.

We conducted several sensitivity analyses (i) to perform a per‐protocol analysis; (ii) to minimise the potential impact of initial dosing differences, only patients who started empagliflozin 10 mg, dapagliflozin at 5 mg or canagliflozin at 100 mg were included; (iii) to account for the possibility that dapagliflozin was more frequently prescribed to higher‐risk patients after its CKD indication expansion, those who started SGLT2is before dapagliflozin's CKD approval were included; (iv) to account for the potential for false‐positive proteinuria associated with UTI, individuals were also censored at the time of UTI onset; (v) without truncation of IPCW weights and (vi) to consider the influence of treating trace proteinuria as negative, the outcome was defined as new‐onset trace or higher proteinuria among patients with negative dipstick results at baseline.

Statistical analyses were performed using SAS (version 9.4 1M7; SAS Institute, Cary, NC, USA) and R software (version 4.4.2; R Foundation for Statistical Computing, Vienna, Austria). Statistical significance was set at *p* < 0.05. This study complied with the STrengthening the Reporting of OBservational studies in Epidemiology (STROBE) statement [37].

## 3 Results

### 3.1 Baseline Characteristics Before and After Weighting

**FIGURE 1:**
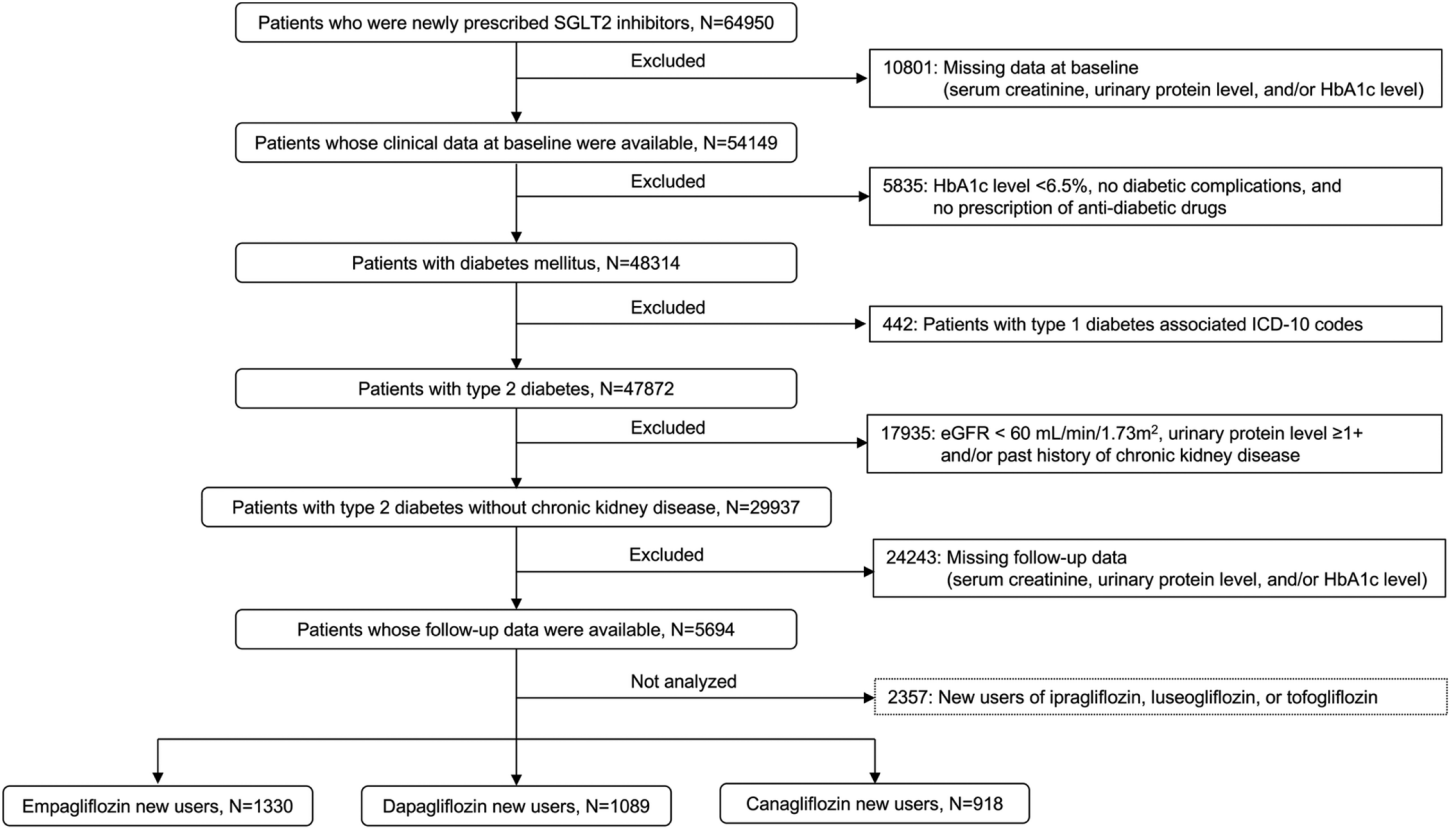
Flowchart of patient selection. eGFR, estimated glomerular filtration rate; HbA1c, haemoglobin A1c; ICD, international classification of diseases; SGLT2, sodium–glucose cotranspoter‐2.

Among the 64 950 new SGLT2is users, 5694 patients with type 2 diabetes and preserved kidney function had both baseline and follow‐up clinical information and were eligible for analysis. Among these patients, 1330, 1089 and 918 were new empagliflozin, dapagliflozin and canagliflozin users, respectively (Figure 1). The baseline characteristics before weighting are summarised in Table S4. The mean age was 67.5 ± 10.3 years, and 60% were men. The mean HbA1c level and eGFR were 7.56% ± 1.32% and 75.0 ± 8.0 mL/min/1.73 m^2^, respectively. During the median follow‐up period of 522 [263–925] days, the mean number of health checkups attended was 1.82, with 52% of patients attending one checkup, 26% attending two checkups and 22% attending three or more checkups. New‐onset proteinuria was observed in 43, 66 and 48 empagliflozin, dapagliflozin and canagliflozin users, respectively.

Table 1 presents the baseline characteristics after weighting. The patients' baseline characteristics were well balanced, with |SMD| < 0.10 (Figures S1–S3). The mean daily dose and proportion of the initial dose for each SGLT2i was as follows: empagliflozin, 10.3 mg (96.5%); dapagliflozin, 5.3 mg (92.6%) and canagliflozin, 99.7 mg (99.1%), indicating that most patients initiated SGLT2 inhibitors at the recommended starting dose.

### 3.2 Weighted Cumulative Incidence of New‐Onset Proteinuria and Risk Assessment

**FIGURE 2:**
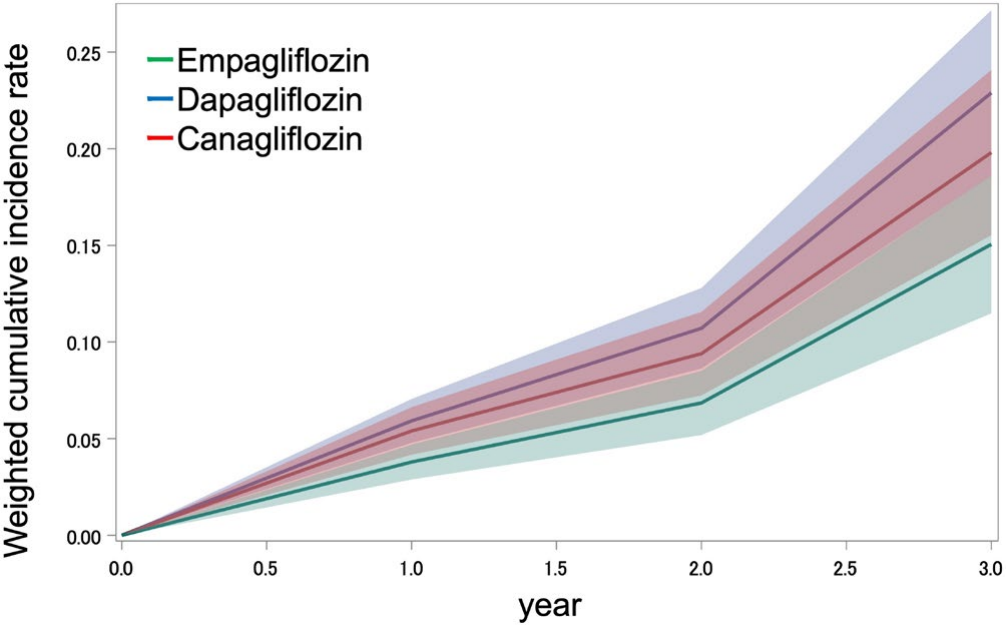
Weighted cumulative incidence rate of new‐onset proteinuria among new users of SGLT2 inhibitors. The shaded area represents the 95% confidence interval. SGLT2, sodium–glucose cotranspoter‐2.

The 3‐year absolute risk of new‐onset proteinuria is illustrated in Figure 2. The risk of new‐onset proteinuria was higher among dapagliflozin new users than among empagliflozin new users (risk difference +7.8%, 95% CI 2.5%–13.2%; HR 1.64, 95% CI 1.15–2.33). The risks were comparable between canagliflozin and empagliflozin users, and between dapagliflozin and canagliflozin users (Table 2).

**TABLE 2:** Risk assessment for new‐onset proteinuria.

	Unweighted	Weighted				
	Events/Total, *n*	Mean follow‐up period, day	3 year‐absolute risk, % (95% CI)	Risk difference vs. empagliflozin, % (95% CI)	Risk difference vs. dapagliflozin, % (95% CI)	HR (95% CI)
Main analysis						
Empagliflozin	43/1330	599	15.1 (11.5–18.6)			Reference
Dapagliflozin	66/1089	685	22.9 (18.6–27.2)	7.8 (2.5–13.2)		1.64 (1.15–2.33)
Canagliflozin	48/918	638	19.8 (15.5–24.1)	4.7 (−0.9–10.4)	−3.1 (−9.3–3.1)	1.35 (0.91–1.98)
Sensitivity analyses						
Per‐protocol analysis^a^						
Empagliflozin	35/1330	387	16.6 (12.4–20.9)			Reference
Dapagliflozin	57/1089	486	25.7 (20.5–30.9)	9.1 (2.6–15.7)		1.75 (1.18–2.60)
Canagliflozin	39/918	405	20.0 (14.6–25.4)	3.4 (−3.5–10.3)	−3.7 (−13.0–1.6)	1.26 (0.79–2.00)
Limiting each daily dose to empagliflozin 10 mg, dapagliflozin 5 mg, and canagliflozin 100 mg						
Empagliflozin	41/1284	597	14.1 (10.6–17.5)			Reference
Dapagliflozin	60/995	657	22.6 (18.1–27.1)	8.5 (2.8–14.1)		1.73 (1.18–2.53)
Canagliflozin	47/909	685	19.1 (14.9–23.3)	5.0 (−0.4–10.5)	−3.4 (−9.5–2.6)	1.39 (0.94–2.07)
Initiation prior to dapagliflozin's CKD indication						
Empagliflozin	32/980	724	12.4 (8.9–16.0)			Reference
Dapagliflozin	47/786	762	19.0 (14.7–23.3)	6.5 (1.0–12.1)		1.61 (1.05–2.47)
Canagliflozin	43/793	767	18.6 (14.5–22.8)	6.2 (0.5–11.8)	−0.3 (−6.2–5.6)	1.58 (1.02–2.44)
Censoring new‐onset UTI^b^						
Empagliflozin	41/1330	340	14.5 (11.0–18.0)			Reference
Dapagliflozin	65/1089	330	22.5 (18.2–26.8)	8.0 (2.7–13.4)		1.68 (1.18–2.41)
Canagliflozin	46/918	395	19.8 (15.5–24.1)	4.7 (−1.0–10.4)	−3.3 (−9.4–2.8)	1.36 (0.91–2.04)
Without truncation of IPCW weights						
Empagliflozin	43/1330	599	15.9 (12.1–19.6)			Reference
Dapagliflozin	66/1089	685	24.0 (19.6–28.5)	8.2 (2.6–13.7)		1.64 (1.16–2.33)
Canagliflozin	48/918	638	20.6 (16.3–25.0)	4.7 (−1.1–10.6)	−3.3 (−9.8–3.0)	1.33 (0.90–1.96)
New‐onset proteinuria defined as trace or higher						
Empagliflozin	90/1144	589	37.1 (32.1–42.2)			Reference
Dapagliflozin	106/915	624	46.7 (41.1–52.2)	9.5 (2.4–16.6)		1.41 (1.08–1.83)
Canagliflozin	94/810	672	46.3 (40.5–52.1)	9.2 (2.0–16.3)	−0.4 (−8.0–7.3)	1.36 (1.05–1.78)

Abbreviations: CI, confidence interval; CKD, chronic kidney disease; HR, hazard ratio; IPCW, inverse probability of censoring weighting; UTI, urinary tract infection.

^a^ A total of 1166 patients receiving empagliflozin, 966 patients receiving dapagliflozin and 776 patients receiving canagliflozin adhered to the study protocol.

^b^ During the observation period, 20 patients receiving empagliflozin, 18 patients receiving dapagliflozin and 20 patients receiving canagliflozin developed urinary tract infections and were censored.

### 3.3 Changes in eGFR and HbA1c Level Among SGLT2is

The decline in eGFR during the first 3 years after initiation of SGLT2is was comparable among the three agents (−2.21, −1.96 and −2.76 mL/min/1.73 m^2^ for empagliflozin, dapagliflozin and canagliflozin, respectively) (Figure S4a). By contrast, the reduction in HbA1c at 3 years after initiation was smaller in dapagliflozin new users than in users of the other agents (−0.53%, −0.25% and −0.68% for empagliflozin, dapagliflozin and canagliflozin, respectively) (Figure S4b).

### 3.4 Subgroup and Sensitivity Analyses

**FIGURE 3:**
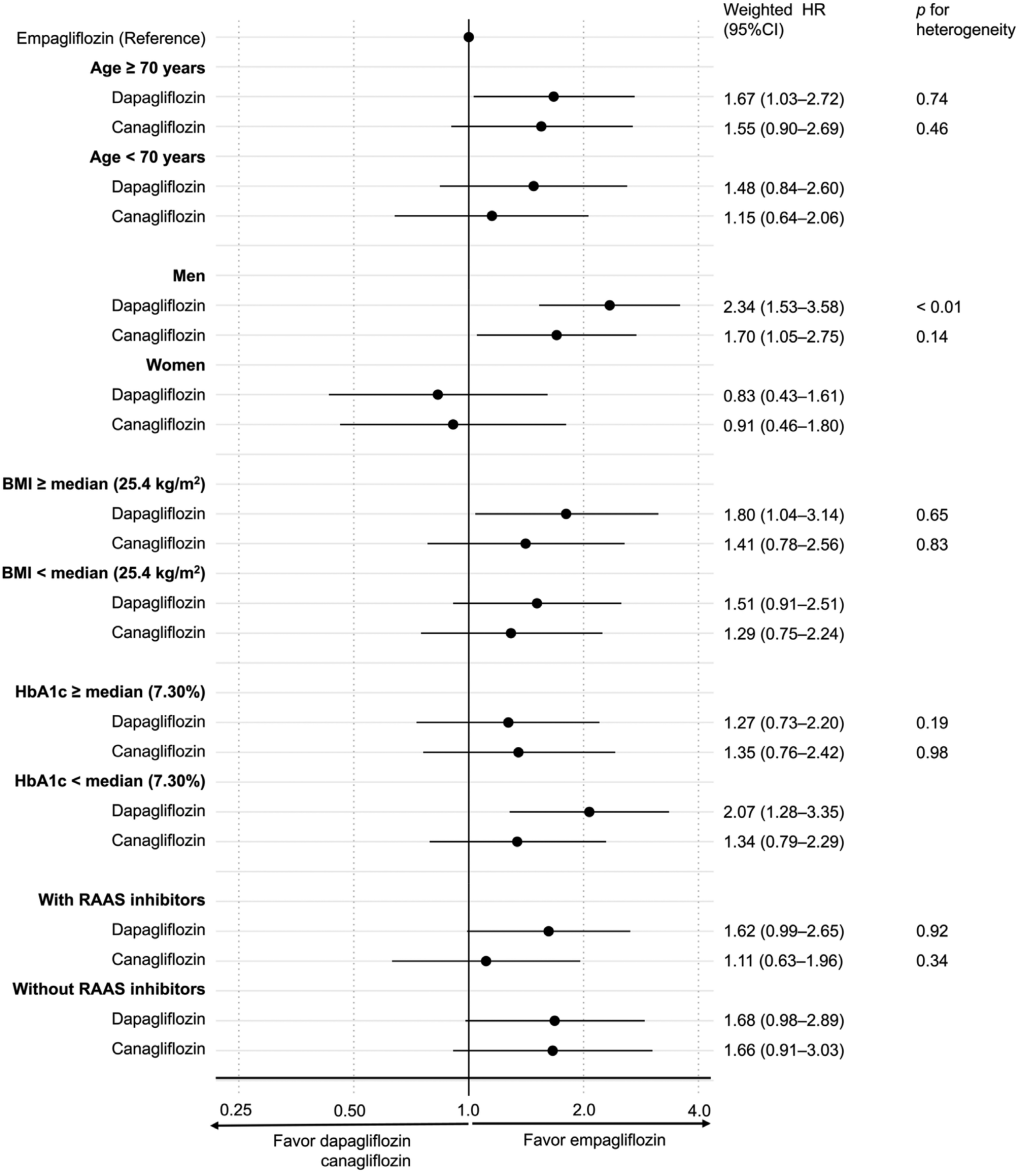
Subgroup analyses of SGLT2 inhibitors' effect on new‐onset proteinuria. BMI, body mass index; CI, confidence interval; HbA1c, haemoglobin A1c; HR, hazard ratio; RAAS, renin–angiotensin–aldosterone system; SGLT2, sodium–glucose cotranspoter‐2.

In the subgroup analysis, significant interactions were observed between the sexes. Among men, dapagliflozin new users had a significantly higher risk of new‐onset proteinuria than empagliflozin new users (HR, 2.34; 95% CI, 1.53–3.58), whereas no increased risk was observed among women (HR, 0.83; 95% CI, 0.43–1.61; *p* for heterogeneity < 0.01). Other factors (age, BMI, HbA1c level and use of RAAS inhibitors) showed no interaction with new‐onset proteinuria among the three agents (Figure 3).

The sensitivity analyses yielded consistent results, supporting the robustness of the primary outcomes (Table 2). In analyses restricted to treatment initiation before the approval of dapagliflozin for CKD and in analyses defining new‐onset proteinuria as trace or higher, new canagliflozin users also showed a higher risk of new‐onset proteinuria than new empagliflozin users.

## 4 Discussion

In this database‐based cohort study, which emulated a target trial comparing new‐onset proteinuria among patients with type 2 diabetes newly prescribed empagliflozin, dapagliflozin, or canagliflozin, some differences in efficacy among these agents were observed. New dapagliflozin users were associated with a higher risk of new‐onset proteinuria than empagliflozin users. This effect was modified by sex and was more pronounced in men. Regarding changes in eGFR and HbA1c 3 years after SGLT2i initiation, the eGFR decline was comparable among the three agents, whereas greater reductions in HbA1c were observed with empagliflozin and canagliflozin than with dapagliflozin.

In this study, empagliflozin was associated with a lower risk of new‐onset proteinuria than dapagliflozin. This finding may be partly attributed to the fact that most new dapagliflozin users (92.6%) initiated treatment at a 5‐mg dose, which is the recommended starting dose for type 2 diabetes in Japan. This hypothesis was further supported by sensitivity analysis restricted to the recommended starting doses (empagliflozin 10 mg and dapagliflozin 5 mg), in which the HR of dapagliflozin was even higher. Thus, the increased risk of new‐onset proteinuria observed with dapagliflozin in the present study may be attributable to the insufficient therapeutic dose of 5‐mg dapagliflozin, and may help address the knowledge gap left by prior large‐scale clinical trials. Although the DECLARE–TIMI 58 trial [38] and DAPA‐CKD trial [39] have demonstrated a significant reduction in new‐onset albuminuria in patients with type 2 diabetes, both trials used dapagliflozin at 10 mg; therefore, the effect of 5‐mg dapagliflozin has remained unclear. Considering our findings, in patients with type 2 diabetes and preserved kidney function initiating dapagliflozin, a prompt dose escalation to 10 mg may be considered for renoprotection if the drug is well tolerated.

The pharmacological differences among the SGLT2is in their selectivity for SGLT2 over SGLT1 may also have contributed to the results of the present study. Empagliflozin demonstrates an approximately 2677‐fold selectivity for SGLT2 over SGLT1, compared with approximately 263‐fold for canagliflozin and 1167‐fold for dapagliflozin, representing an approximately 10‐fold variation among the three agents [40, 41, 42]. In the sensitivity analysis restricted to patients who initiated treatment before dapagliflozin received an indication for CKD, canagliflozin was also associated with a higher risk of new‐onset proteinuria compared with empagliflozin. The finding that only dapagliflozin showed a significant association with new‐onset proteinuria risk in the primary analysis may reflect the preferential prescription of dapagliflozin to patients at higher renal risk after its approval for CKD.

In the analysis of changes in HbA1c levels, empagliflozin and canagliflozin were associated with greater reductions in HbA1c levels than dapagliflozin. Pharmacodynamic studies comparing urinary glucose excretion have shown that empagliflozin 10 mg and canagliflozin 100 mg achieve approximately 80 g/day of urinary glucose excretion on the first day of treatment, whereas dapagliflozin achieves approximately 70 g/day even at a 10‐mg dose [43, 44, 45]. Although direct comparisons across these studies are limited by differences in study populations and designs, the findings from these pharmacodynamic studies are consistent with our observed differences in HbA1c reduction. By contrast, the decline in eGFR from baseline was comparable among them. These findings were consistent with that of a study using a large‐scale real‐world dataset from Japan, which has reported no significant differences in eGFR decline over a mean observation period of 773 days among patients treated with canagliflozin, dapagliflozin, empagliflozin or other SGLT2is [46]. The reduction in proteinuria induced by SGLT2is is primarily attributed to the attenuation of glomerular hyperfiltration through restoration of the tubuloglomerular feedback mechanism [47, 48, 49]. In patients with type 2 diabetes and preserved kidney function, hyperfiltration typically precedes the development of proteinuria, followed by progressive glomerulosclerosis and subsequent declines in GFR. This suggests a temporal lag between changes in proteinuria and measurable declines in eGFR [3]. Therefore, the follow‐up duration of the present study may have been insufficient to determine whether the observed differences in the risk of new‐onset proteinuria translate into differential long‐term trajectories of eGFR decline. Long‐term follow‐up is required to clarify the impact of these differences on renal function.

## 5 Limitations

This study has some limitations. First, owing to the retrospective design, even with TTE, residual confounding factors such as diabetes duration cannot be completely ruled out [50]. Empagliflozin may have been preferentially prescribed earlier in the course of type 2 diabetes, as SGLT2is were already widespread at the time of its introduction, potentially introducing selection bias. Although appropriately designed studies based on the TTE framework produce estimates consistent with RCTs [51], further confirmation through future randomised trials or studies in different populations is required. Second, the urinalysis results were based on a single measurement, and the results of qualitative dipstick tests for urinary proteins were used. The evaluation of urinary albumin excretion is clinically important in patients with type 2 diabetes. The use of dipstick proteinuria (≥ 1+) in this study may have overlooked earlier changes, including microalbuminuria. Third, restricting the cohort to individuals who underwent health checkups may have introduced a self‐selection bias, potentially selecting a population with relatively preserved kidney function. The eGFR decline observed in this study was more gradual than that reported in large clinical trials [7, 52, 53]. The evaluation of populations at a higher risk of kidney function decline is important for confirming the applicability of our findings. Fourth, in the databases used, individual records were terminated, and a new patient identifier was assigned when insurance coverage changes, which may have resulted in duplicate individuals across databases. However, owing to the new‐user design with a 180‐day washout period, duplication would be unlikely unless treatment was interrupted for > 180 days before the insurance change; therefore, its impact is likely limited. Fifth, in this study, disease definitions relied on ICD‐10 codes in the Japanese claims data, which may be subject to misclassification. As summarised in a prior review [15], this represents an inherent and unavoidable limitation of database studies that use administrative data from Japan. Finally, the database used in this study does not cover all public health insurance systems in Japan and underrepresents working‐age individuals. Thus, a moderate proportion of the study population comprised older individuals, resulting in a relatively higher mean age of the cohort. Therefore, further studies involving broader age ranges are required.

## 6 Conclusions

Among patients with type 2 diabetes and preserved kidney function, empagliflozin may be associated with a lower risk of new‐onset proteinuria than dapagliflozin, potentially reflecting differences in dosing. SGLT2is selection should be individualised based on patient characteristics and dose optimization for the management of type 2 diabetes.

## Author Contributions

Michihiro Satoh had full access to all data in the study and takes responsibility for the integrity of the data and the accuracy of the data analysis. Conceptualization: Hiroki Nobayashi and Michihiro Satoh. Methodology: Hiroki Nobayashi and Michihiro Satoh. Formal analysis and investigation: Hiroki Nobayashi, Michihiro Satoh and Hirohito Metoki. Writing – original draft preparation: Hiroki Nobayashi and Michihiro Satoh. Writing – review and editing: Takuo Hirose, Shingo Nakayama, Yutaro Iwabe, Hideaki Hashimoto, Takahisa Murakami, Kouji Okada, Takefumi Mori and Hirohito Metoki.

## Funding

This study was supported by Grants for Scientific Research (KAKENHI) (Grant Number: JP21K10478 and JP25K02854) from the Japan Society for the Promotion of Science (JSPS), Japan; grants from the Medical Research Encouragement Prize of the Japan Medical Association, Medical Association, Japan; and Kowa Life Science Foundation, Japan.

## Conflicts of Interest

Michihiro Satoh received academic support from the Kowa Life Science Foundation, funded by Kowa Co. Ltd. and Bayer Yakuhin Co. Ltd. Michihiro Satoh received lectures from FUJIYAKUHIN Co. Ltd. The Division of Public Health, Hygiene and Epidemiology (Tohoku Medical and Pharmaceutical University) has received research grants from Astellas Pharma Inc., Daiichi Sankyo Company Ltd., Baxter International Inc. and Bayer Co. Ltd.

## Supporting information


**Data S1:** dom70625‐sup‐0001‐supinfo_1.docx.

**Data S2:** dom70625‐sup‐0002‐supinfo_2.docx.

## Data Availability

Data supporting the study findings are available from DeSc Healthcare Inc. Restrictions apply to the availability of the data, which were used under a licence for this study. The data are available from the authors with permission from DeSc Healthcare Inc.
