# Supplementary material for: Comparison of SGLT2 Inhibitors for New‐Onset Proteinuria Risk in Patients With Type 2 Diabetes and Preserved Kidney Function
Source: Diabetes Obes Metab. 2026 Mar 9;28(5):4314–25. doi: 10.1111/dom.70625 (PMC13071193; doi:10.1111/dom.70625)
Supplement: Supplementary file 1 — Data S1: dom70625‐sup‐0001‐supinfo_1.docx. [file DOM-28-4314-s001.docx]

# **Supporting Information**

**Table S1**: Comparison of target and emulated trials

**Table S2**: Classification of antidiabetic and antihypertensive drugs according to ATC codes

**Table S3**: Disease definitions according to ICD-10 codes

**Table S4**: Characteristics of study population before weighting

**Figure S1**: Love plot of absolute standardized mean differences between empagliflozin and dapagliflozin before and after weighting

**Figure S2**: Love plot of absolute standardized mean differences between empagliflozin and canagliflozin before and after weighting

**Figure S3**: Love plot of absolute standardized mean differences before and after weighting between dapagliflozin and canagliflozin

**Figure S4**: Changes in estimated glomerular filtration rate and HbA1c levels among the three sodium-glucose cotranspoter-2 inhibitors.

# **Table S1. Comparison of target and emulated trials**

| **Component** | **Target trial** | **Emulated trial** |
| --- | --- | --- |
| **Eligibility criteria** | - Patients with type 2 diabetes - eGFR ≥60 mL/min/1.73m^2^ and normoalbuminuria | - Hemoglobin A1c level ≥6.5% and/or use of anti-diabetic drugs, and/or the presence of an ICD-10 code for non-insulin-dependent diabetes mellitus - Without type 1 diabetes associated ICD-10 codes - eGFR ≥60 mL/min/1.73 m^2^, a urine protein level < 1+ as determined by qualitative dipstick testing, and the absence of ICD-10 codes for CKD |
| **Treatment strategies** | Initiation of canagliflozin, dapagliflozin, or empagliflozin | Same as target trial |
| **Assignment procedure** | Participants are randomly assigned to one of the three SGLT2 inhibitors with 1:1:1 ratio | Randomization was emulated through the use of baseline covariates to derive IPTW, which were applied to achieve balance across treatment strategies. |
| **Follow-up start** | At randomization | At the day of treatment assignment |
| **Follow-up end** | At the occurrence of outcomes or loss to follow-up, whichever occurs first | At the earliest of the occurrence of the outcome, the last health checkup date when no subsequent checkup was recorded for more than 2 years after the previous visit, or the final health checkup date within the observation period |
| **Outcome** | New onset of micro- or macro- albuminuria | New onset of urine protein level ≥1+ as determined by qualitative dipstick testing |
| **Causal contrast of interest** | Intention-to-treat effect | Same as target trial |
| **Analysis plan** | The intention-to-treat effect will be estimated via Cox proportional hazard regression among individuals assigned to each treatment strategy. The analyses will be conducted for the whole population and subgroups of each category (age, sex, body mass index, and hemoglobin A1c level) | The intention-to-treat effect was estimated using IPTW derived from relevant pre-baseline covariates to emulate randomization, while loss to follow-up due to health check-up attendance was accounted for using IPCW. The weighted analyses were conducted using pooled logistic regression models to estimate hazard ratios and absolute risks. Analyses were performed in the overall population and in prespecified subgroups defined by age, sex, body mass index, hemoglobin A1c level, and use of RAAS inhibitors. |

CKD, chronic kidney disease; eGFR, estimated glomerular filtration rate; ICD, International Classification of Diseases; IPCW, inverse probability of censoring weights; IPTW, inverse probability of treatment weighting; RAAS, renin–angiotensin–aldosterone system; SGLT2, sodium-glucose cotransporter 2.

# **Table S2.** **Classification of anti-diabetic and anti-hypertensive drugs by ATC codes**

| **Drugs** | **ATC codes** | | | |
| --- | --- | --- | --- | --- |
| **Anti-diabetic drugs** |  |  |  |  |
| Dapagliflozin | A10BK01 |  |  |  |
| Canagliflozin | A10BK02 |  |  |  |
| Empagliflozin | A10BK03 |  |  |  |
| Ipragliflozin | A10BK05 |  |  |  |
| Luseogliflozin | A10BK07 |  |  |  |
| Tofogliflozin | A10BK |  |  |  |
| Biguanide | A10BA |  |  |  |
| DPP-4i | A10BH |  |  |  |
| Sulfonylurea | A10BB |  |  |  |
| Glinide | A10BX |  |  |  |
| α-Glucosidase inhibitor | A10BF |  |  |  |
| Thiazolidinedione | A10BG |  |  |  |
| GLP-1RA | A10BJ | A10BX |  |  |
| Insulin | A10AB | A10AC | A10AD | A10AE |
| **Anti-hypertensive drugs** |  |  |  |  |
| Dihydropyridine derivative | C08CA |  |  |  |
| RASI | C09AA | C09CA |  |  |
| MRA | C03DA | C03DB |  |  |
| Thiazide diuretics | C03AA | C03BA |  |  |
| Loop diuretics | C03CA01 |  |  |  |
| β-αβ blockers | C07AA | C07AB | C07AG |  |
| α-Blocker | C02CA | G04CA03 |  |  |
| **Lipid-lowering drugs** |  |  |  |  |
| HMG CoA reductase inhibitors | C10AA |  |  |  |
| Fibrates | C10AB |  |  |  |
| **Uric acid-lowering drugs** |  |  |  |  |
| Preparations inhibiting uric acid production | M04AA |  |  |  |
| Preparations increasing uric acid excretion | M04AB |  |  |  |

ATC, Anatomical Therapeutic Chemical; DPP-4i, dipeptidyl peptidase 4 inhibitor; GLP-1RA, glucagon-like peptide-1 receptor agonist; MRA, mineralocorticoid receptor antagonist; RASI, renin–angiotensin system inhibitor.

# **Table S3. Disease definitions by ICD-10 codes**

| Disease group | Disease | Codes |
| --- | --- | --- |
| Cerebrovascular disease | Subarachnoid hemorrhage | I601–I609 |
|  | Intracerebral hemorrhage | I610–I616, I618, I619 |
|  | Cerebral infarction | I630–I636, I638, I639 |
| Ischemic heart disease | Angina pectoris | I200, I201, I208, I209 |
|  | Acute myocardial infarction | I211–I214, I219 |
|  | Subsequent myocardial infarction | I220, I221, I228, I229 |
|  | Complications following myocardial infarction | I230–I236, I238 |
|  | Hypertensive heart disease with heart failure | I110, I130, I132 |
|  | Heart failure | I500, I501, I509 |
| Chronic kidney disease |  | N180–N185, N188, N189 |
| Insulin-dependent diabetes mellitus |  | E10, E100–E109 |
| Non-insulin-dependent diabetes mellitus |  | E11, E110–E119 |
| Urinary tract infection | Pyelonephritis | N10–N12 |
|  | Lower urinary tract infection | N30, N34, N390 |

# **Table S4. Participant characteristics before weighting**

|  | Unweighted | | | | | |
| --- | --- | --- | --- | --- | --- | --- |
|  | Empagliflozin  *N* = 1330 | Dapagliflozin  *N* = 1089 | Canagliflozin  *N* = 918 | \|SMD\| Empagliflozin vs. dapagliflozin | \|SMD\| Empagliflozin vs. canagliflozin | \|SMD\| Dapagliflozin vs. canagliflozin |
| Age, year (SD) | 67.3 (10.3) | 68.5 (10.3) | 66.8 (10.4) | 0.12 | 0.05 | 0.17 |
| Later-stage  Elderly Healthcare System, n (%) | 314 (24) | 332 (30) | 214 (23) | 0.16 | 0.01 | 0.16 |
| Male, n (%) | 781 (59) | 645 (59) | 575 (59) | 0.10 | 0.08 | 0.07 |
| BMI, kg/m^2^ (SD)* | 25.9 (4.1) | 25.7 (4.0) | 26.0 (4.2) | 0.04 | 0.03 | 0.08 |
| SBP, mmHg (SD) | 132.1 (15.8) | 132.1 (16.1) | 133.0 (15.9) | 0.00 | 0.06 | 0.06 |
| Drinker, n (%)* | 207 (19) | 171 (20) | 143 (18) | 0.17 | 0.05 | 0.23 |
| Smoker, n (%)* | 207 (18) | 196 (18) | 154 (19) | 0.15 | 0.03 | 0.19 |
| LDL-cholesterol, mmol/L (SD) | 2.96 (0.80) | 2.94 (0.79) | 2.97 (0.82) | 0.03 | 0.01 | 0.04 |
| HbA1c, % (SD) | 7.49 (1.20) | 7.60 (1.37) | 7.63 (1.41) | 0.09 | 0.11 | 0.02 |
| Trace proteinuria, n (%) | 186 (14) | 174 (16) | 108 (12) | 0.06 | 0.07 | 0.12 |
| eGFR, mL/min/1.73m^2^ (SD) | 75.0 (8.0) | 74.6 (7.8) | 75.6 (8.0) | 0.05 | 0.08 | 0.13 |
| Self-reported past history, n (%) |  |  |  |  |  |  |
| Stroke* | 50 (4) | 35 (4) | 22 (3) | 0.20 | 0.01 | 0.19 |
| Heart disease* | 138 (12) | 100 (11) | 80 (10) | 0.19 | 0.00 | 0.19 |
| ICD-based past history, n (%) |  |  |  |  |  |  |
| Cerebrovascular disease | 122 (9) | 113 (10) | 60 (7) | 0.04 | 0.09 | 0.14 |
| Ischemic heart disease | 285 (21) | 221 (20) | 144 (16) | 0.03 | 0.15 | 0.12 |
| Heart failure | 246 (19) | 216 (20) | 119 (13) | 0.03 | 0.15 | 0.18 |
| Prescription before dapagliflozin’s heart failure indication | 803 (60) | 595 (55) | 655 (71) | 0.12 | 0.23 | 0.35 |
| Anti-hypertensive drugs, n (%) |  |  |  |  |  |  |
| Dihydropyridine derivative | 525 (39) | 463 (43) | 364 (40) | 0.06 | 0.00 | 0.06 |
| RASI | 588 (44) | 449 (41) | 393 (43) | 0.06 | 0.03 | 0.03 |
| MRA | 40 (3) | 43 (4) | 15 (2) | 0.05 | 0.09 | 0.14 |
| Thiazide diuretics | 67 (5) | 56 (5) | 56 (6) | 0.00 | 0.05 | 0.04 |
| Loop diuretics | 61 (5) | 45 (4) | 16 (2) | 0.02 | 0.16 | 0.14 |
| β-αβ blockers | 166 (12) | 157 (14) | 84 (9) | 0.06 | 0.11 | 0.16 |
| α blockers | 14 (1) | 7 (1) | 11 (1) | 0.04 | 0.01 | 0.06 |

| **Table S4. Participant characteristics before weighting (continued)** | | | | | | |
| --- | --- | --- | --- | --- | --- | --- |
|  | Unweighted | | | | | |
|  | Empagliflozin  *N* = 1330 | Dapagliflozin  *N* = 1089 | Canagliflozin  *N* = 918 | \|SMD\| Empagliflozin vs. dapagliflozin | \|SMD\| Empagliflozin vs. canagliflozin | \|SMD\| Dapagliflozin vs. canagliflozin |
| Lipid-lowering drugs, n (%) |  |  |  |  |  |  |
| HMG CoA reductase inhibitors | 671 (50) | 547 (50) | 447 (49) | 0.00 | 0.04 | 0.03 |
| Fibrates | 72 (5) | 60 (6) | 69 (8) | 0.00 | 0.09 | 0.08 |
| Uric acid-lowering drugs, n (%) |  |  |  |  |  |  |
| Preparations inhibiting uric acid production | 92 (7) | 75 (7) | 74 (8) | 0.00 | 0.04 | 0.04 |
| Preparations increasing uric acid excretion | 10 (1) | 9 (1) | 7 (1) | 0.01 | 0.00 | 0.01 |
| Dose of SGLT2i, mg/day (SD) | 10.2 (2.3) | 5.4 (1.4) | 99.7 (5.7) |  |  |  |
| Anti-diabetic drugs, n (%) |  |  |  |  |  |  |
| Biguanide | 600 (45) | 442 (41) | 385 (42) | 0.09 | 0.06 | 0.03 |
| DPP-4i | 579 (44) | 579 (53) | 634 (69) | 0.19 | 0.53 | 0.33 |
| Sulfonylurea | 253 (19) | 205 (19) | 171 (19) | 0.01 | 0.01 | 0.01 |
| Glinide | 74 (6) | 53 (5) | 55 (6) | 0.03 | 0.02 | 0.05 |
| α-Glucosidase inhibitors | 144 (11) | 137 (13) | 108 (12) | 0.05 | 0.03 | 0.02 |
| Thiazolidinedione | 98 (7) | 66 (6) | 81 (9) | 0.05 | 0.05 | 0.11 |
| GLP-1RA | 27 (2) | 19 (2) | 7 (1) | 0.02 | 0.11 | 0.09 |
| Insulin | 99 (7) | 62 (6) | 53 (6) | 0.07 | 0.07 | 0.00 |

BMI, body mass index; DPP-4i, dipeptidyl peptidase 4 inhibitor; eGFR, estimated glomerular filtration rate; GLP-1RA, glucagon-like peptide-1 receptor agonist; HbA1c, hemoglobin A1c; ICD, international classification of diseases; LDL-C, low-density lipoprotein cholesterol; MRA, mineralocorticoid receptor antagonist; RASI, renin–angiotensin system inhibitor; SBP, systolic blood pressure; SGLT2i, sodium-glucose cotranspoter-2 inhibitor; SMD, standardized mean difference.

*Missing data:
BMI, 1 for the dapagliflozin group
Drinker, 217 empagliflozin, 253 dapagliflozin, 217 canagliflozin;
 smoker, 177 empagliflozin, 215 dapagliflozin, 106 canagliflozin;
 stroke, 143 empagliflozin, 198 dapagliflozin, and 107 canagliflozin;
 heart disease, 142 empagliflozin, 198 dapagliflozin, and 107 canagliflozin.

# **Figure S1. Love plot of absolute standardized mean differences before and after weighting between empagliflozin and dapagliflozin**


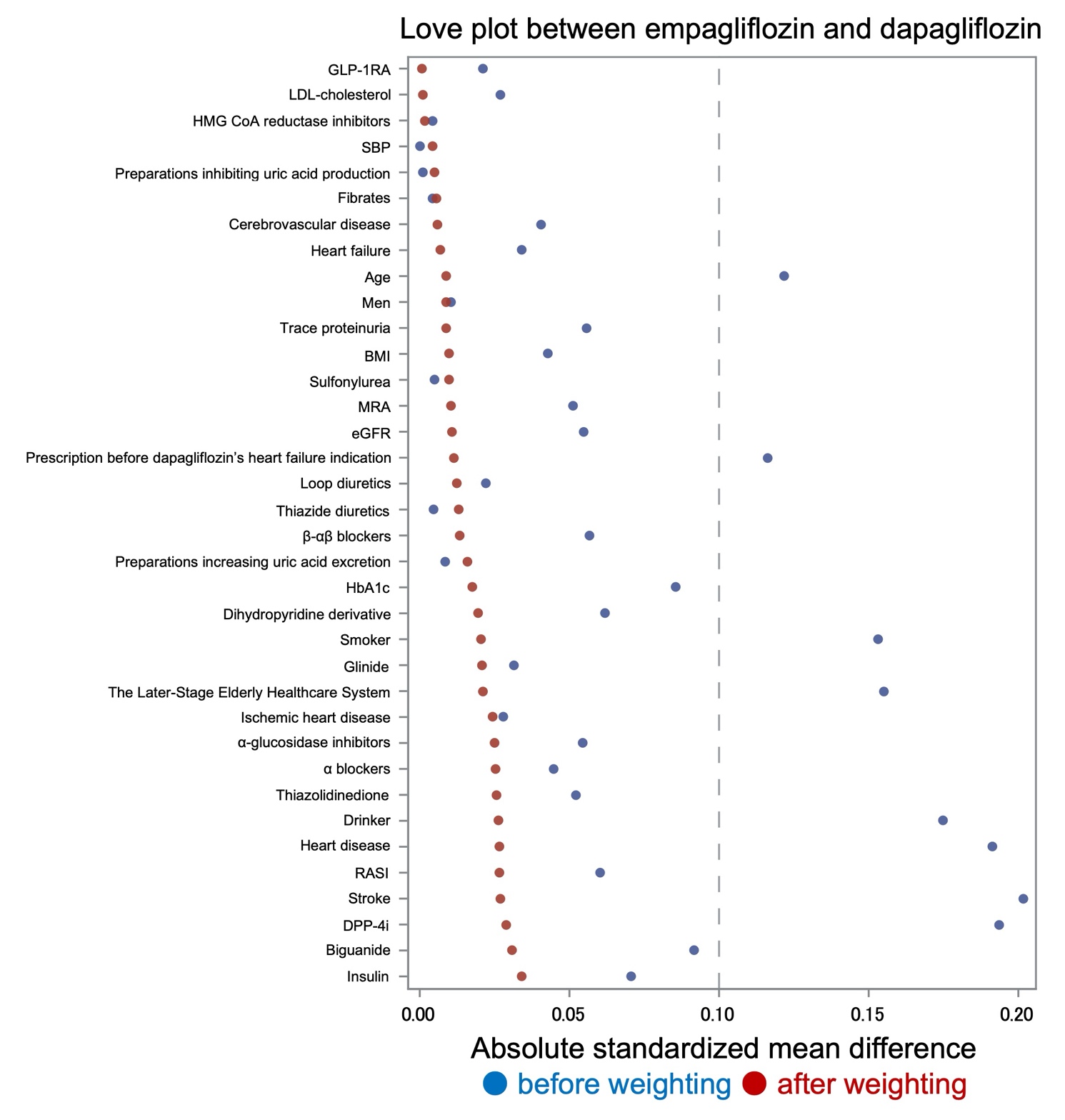


BMI, body mass index; DPP-4i, dipeptidyl peptidase 4 inhibitor; eGFR, estimated glomerular filtration rate; GLP-1RA, glucagon-like peptide-1 receptor agonist; HbA1c, hemoglobin A1c; LDL-C, low-density lipoprotein cholesterol; MRA, mineralocorticoid receptor antagonist; RASI, renin–angiotensin system inhibitor; SBP, systolic blood pressure.

# **Figure S2. Love plot of absolute standardized mean differences before and after weighting between empagliflozin and canagliflozin**


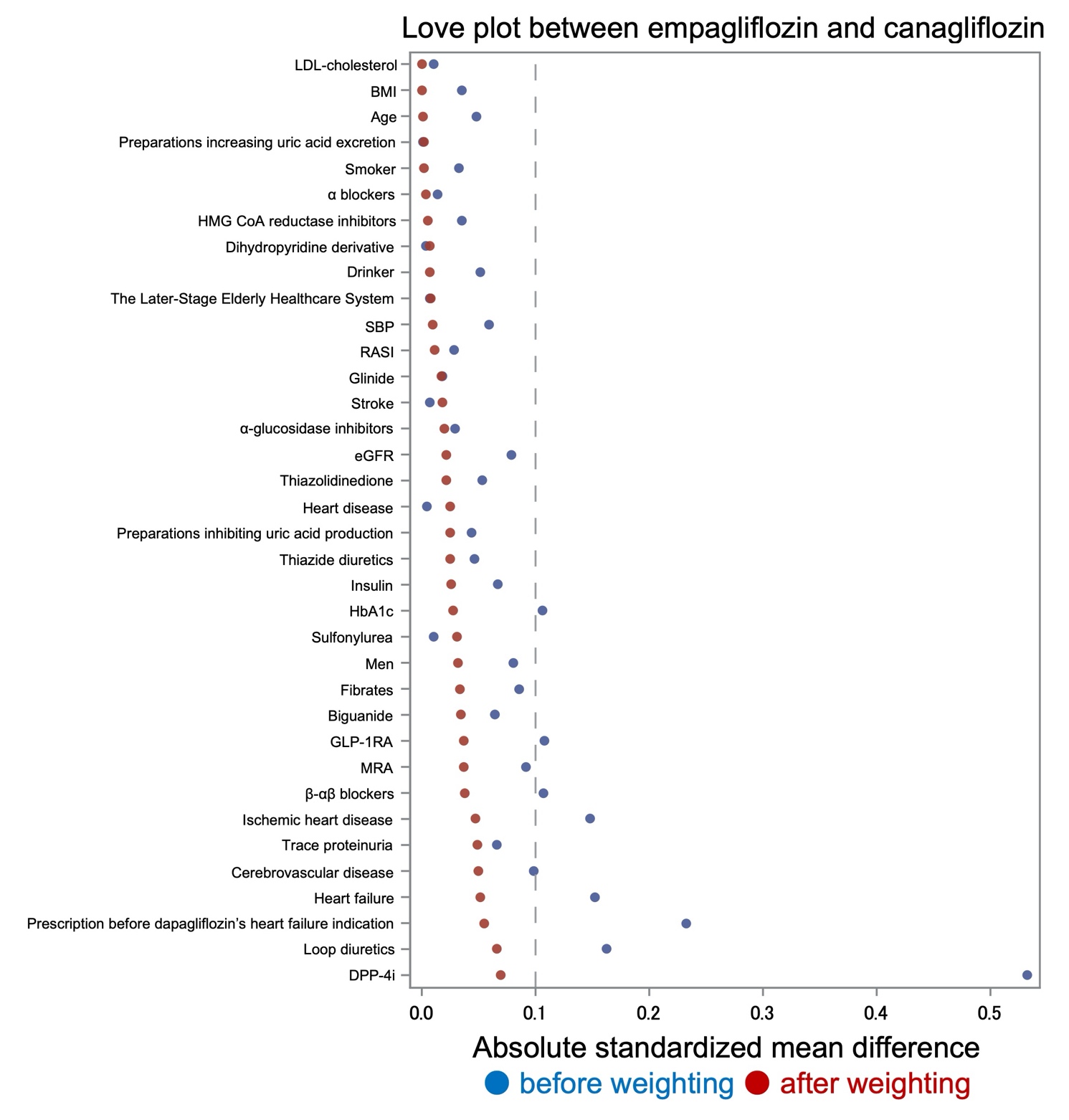


BMI, body mass index; DPP-4i, dipeptidyl peptidase 4 inhibitor; eGFR, estimated glomerular filtration rate; GLP-1RA, glucagon-like peptide-1 receptor agonist; HbA1c, hemoglobin A1c; LDL-C, low-density lipoprotein cholesterol; MRA, mineralocorticoid receptor antagonist; RASI, renin–angiotensin system inhibitor; SBP, systolic blood pressure.

# **Figure S3. Love plot of absolute standardized mean differences before and after weighting between dapagliflozin and canagliflozin**


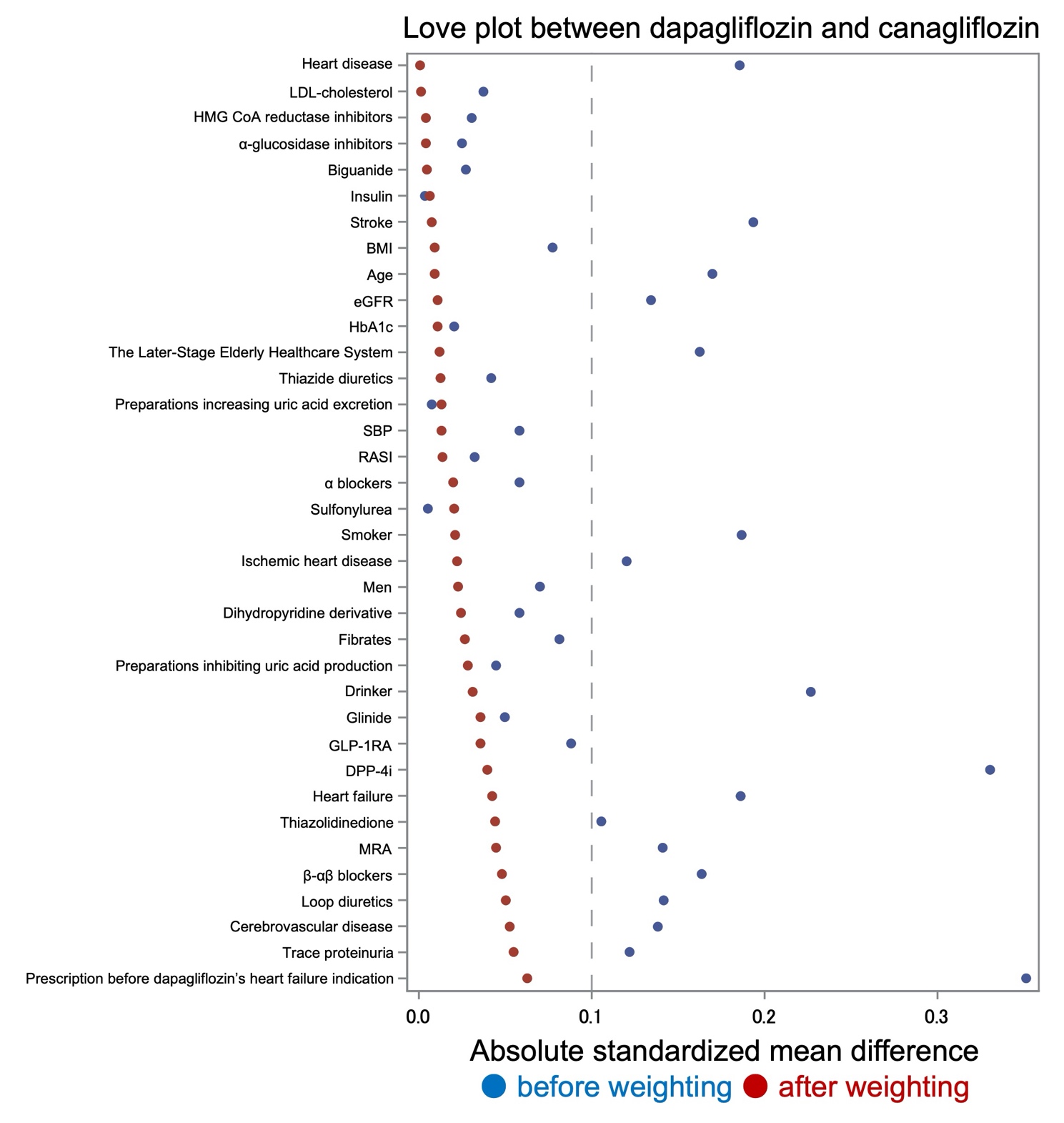


BMI, body mass index; DPP-4i, dipeptidyl peptidase 4 inhibitor; eGFR, estimated glomerular filtration rate; GLP-1RA, glucagon-like peptide-1 receptor agonist; HbA1c, hemoglobin A1c; LDL-C, low-density lipoprotein cholesterol; MRA, mineralocorticoid receptor antagonist; RASI, renin–angiotensin system inhibitor; SBP, systolic blood pressure.

# **Figure S4. Weighted change in eGFR and HbA1c level among three SGLT2 inhibitors**


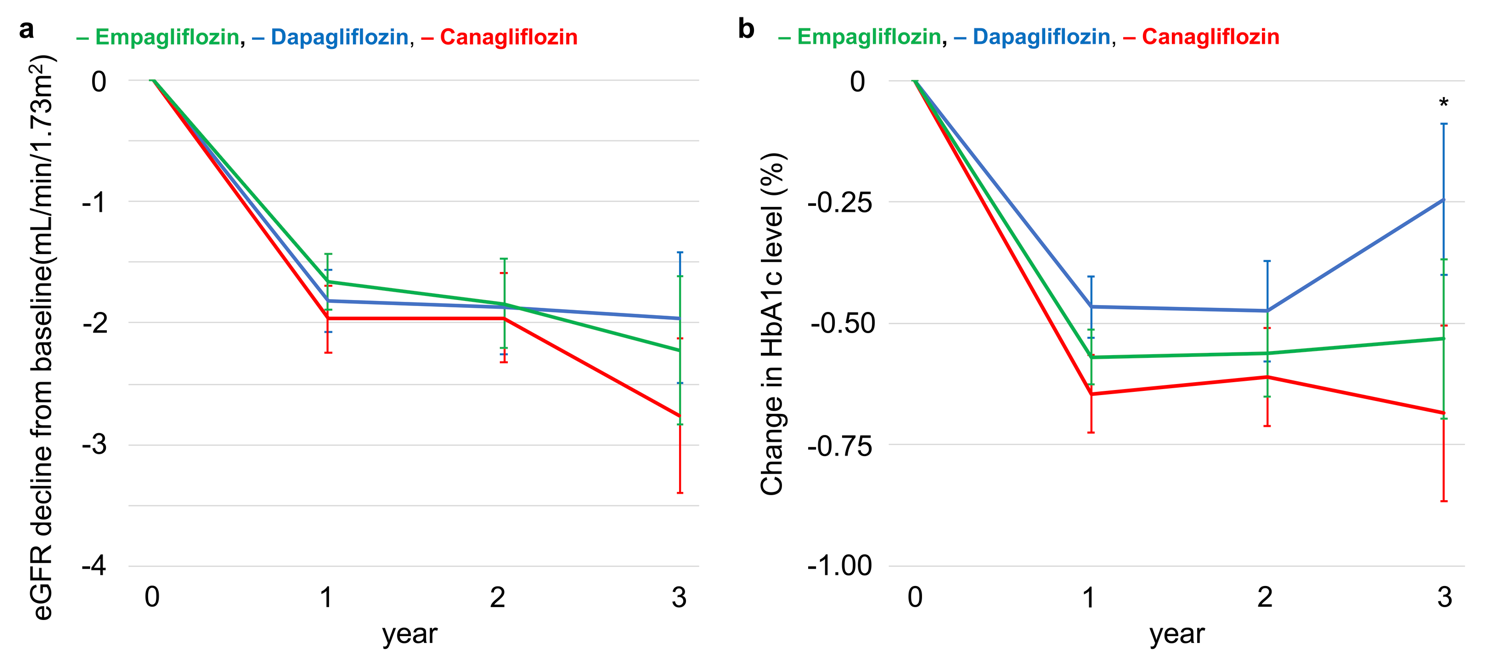


a, Changes in the estimated glomerular filtration rate (eGFR) were estimated and compared using weighted analyses based on annually measured eGFR values. Annual changes were calculated from the mean estimates and 95% confidence intervals obtained from 1,000 bootstrap resamples. No significant differences in eGFR decline were observed between the canagliflozin, dapagliflozin, and empagliflozin groups at any time point.

b, Changes in hemoglobin A1c (HbA1c) levels were estimated and compared using weighted analyses based on annually measured HbA1c values. Annual changes were calculated from mean estimates and 95% confidence intervals derived from 1,000 bootstrap resamples. At year 3, HbA1c reductions were significantly greater with canagliflozin and empagliflozin than with dapagliflozin (empagliflozin vs dapagliflozin: −0.29%, 95% CI −0.51 to −0.06; canagliflozin vs dapagliflozin: −0.44%, 95% CI −0.68 to −0.20).

The green, blue, and red lines represent empagliflozin, dapagliflozin, and canagliflozin, respectively. Points indicate the estimated means, and error bars represent 95% confidence intervals. CI, confidence interval; eGFR, estimated glomerular filtration rate; HbA1c, hemoglobin A1c.
